# Is CD19-directed chimeric antigen receptor T cell therapy a smart strategy to combat central nervous system lymphoma?

**DOI:** 10.3389/fonc.2022.1082235

**Published:** 2023-01-05

**Authors:** Kotaro Miyao, Hirofumi Yokota, R. Leo Sakemura

**Affiliations:** ^1^ Department of Hematology and Oncology, Anjo Kosei Hospital, Anjo, Japan; ^2^ Department of Hematology and Oncology, Nagoya University Graduate School of Medicine, Nagoya, Japan; ^3^ T Cell Engineering, Mayo Clinic, Rochester, MN, United States; ^4^ Division of Hematology, Mayo Clinic, Rochester, MN, United States

**Keywords:** CAR-T cell, PCNSL, CNS, CNS penetration, local administration

## Abstract

Primary central nervous system lymphoma (PCNSL) is a rare form and aggressive type of diffuse large B-cell lymphoma (DLBCL) that occurs in both immunocompetent and immunocompromised adults. While adding rituximab to chemotherapeutic regimens resulted in dramatic improvement in both progression-free survival and overall survival in patients with non-central nervous system (CNS) DLBCL, the outcomes of PCNSL are generally poor due to the immune-privileged tumor microenvironment or suboptimal delivery of systemic agents into tumor tissues. Therefore, more effective therapy for PCNSL generally requires systemic therapy with sufficient CNS penetration, including high-dose intravenous methotrexate with rituximab or high-dose chemotherapy followed by autologous stem cell transplantation. However, overall survival is usually inferior in comparison to non-CNS lymphomas, and treatment options are limited for elderly patients or patients with relapsed/refractory disease. Chimeric antigen receptor T (CAR-T) cell therapy has emerged as a cutting-edge cancer therapy, which led to recent FDA approvals for patients with B-cell malignancies and multiple myeloma. Although CAR-T cell therapy in patients with PCNSL demonstrated promising results without significant toxicities in some small cohorts, most cases of PCNSL are excluded from the pivotal CAR-T cell trials due to the concerns of neurotoxicity after CAR-T cell infusion. In this review, we will provide an overview of PCNSL and highlight current approaches, resistance mechanisms, and future perspectives of CAR-T cell therapy in patients with PCNSL.

## Introduction

Primary central nervous system lymphoma (PCNSL) is a rare malignancy with an annual incidence of 4-7 per one million people in the United States (1, 2). According to the World Health Organization classification, PCNSL is categorized as a sub-type of aggressive non-Hodgkin lymphoma (NHL) which develops in the central nervous system (CNS) (3–5). Unlike most other CNS malignancies, PCNSL is often responsive to chemotherapy and/or radiation therapy. However, outcomes are inferior compared to non-CNS lymphomas, with a median overall survival of 1.5 months when untreated (6). Furthermore, the prognosis for relapsed and/or refractory (R/R) PCNSL is significantly worse, especially when patients are not eligible for autologous stem cell transplantation (ASCT) or relapse after ASCT.

Chimeric antigen receptor T (CAR-T) cell therapy has emerged as a potent and potentially curative therapy in hematological malignancies (7–9). Pivotal clinical trials of CD19-directed CAR-T (CART19) cell therapy demonstrated unprecedented results in non-CNS B-cell lymphomas and acute lymphoblastic leukemia (ALL), leading to several FDA approvals of CART19 cell products (10–21). Although novel therapeutic approaches have improved overall response and survival in non-CNS lymphomas, the application of CAR-T cell therapy in PCNSL is not yet established. Nevertheless, recent clinical data have suggested efficacy and tolerable safety of CART19 cell therapy in secondary CNS lymphoma as well as PCNSL (22–25).

In this review article, we will focus on CAR-T therapy in patients with PCNSL, highlighting outcomes of reported cases and offering future perspectives to overcome resistance and improve CAR-T cell activity within the CNS.

## Overview of PCNSL

PCNSL is a highly aggressive and rare NHL which includes lesions in the brain, spinal cord, cerebrospinal fluid (CSF), or eyes (3). Although rare, the incidence of PCNSL has been increasing in recent years, especially in patients older than 65 (1). Patients with immunosuppressive conditions such as human immunodeficiency virus 1 (HIV-1) infections can develop PCNSL, but the etiology and treatment are separate from immunocompetent patients with PCNSL (26). In this review article, we will mainly discuss PCNSL developed in immunocompetent patients and briefly review PCNSL in patients with HIV-1 infection.

Frontline treatment strategies of PCNSL have been improving. In the past, whole brain radiotherapy (WBRT) alone was the standard therapy for decades. WBRT showed a strong initial response, but the relapse rate was high and showed poor survival (27). Chemotherapy alone, particularly high-dose methotrexate (MTX), showed better efficacy and less neurotoxicity than WBRT. For example, prospective analyses of treatment strategies in PCNSL revealed the safety and efficacy of high-dose MTX-based induction therapy and cytarabine (AraC)-based consolidation even in patients older than 60 (28, 29). These findings demonstrated the importance of high-dose MTX-based induction and other consolidation therapy. WBRT is also a selective consolidation approach especially for patients who are not eligible for high dose chemotherapy followed by ASCT. Some trials reported that the combination therapy of chemotherapy and WBRT achieved higher response rates and lower toxicity compared with chemotherapy alone. In a prospective multicenter study, patients underwent rituximab, MTX, vincristine, and procarbazine (R-MVP) and demonstrated 2-year overall survival (OS) of 67%, and no treatment-related neurotoxicity was observed (30). However, there are concerns about an increased risk of neurotoxicity after WBRT in long-term survivors (31). Therefore, dose reduction of WBRT should be considered when used as consolidation therapy. An alternate approach for consolidation therapy to improve efficacy without an increased risk of neurotoxicity is high dose chemotherapy followed by ASCT for eligible patients. ANOCEF-GOELAMS Randomized Phase 2 PRECIS Study reported that ASCT showed superior event free survival after consolidations to WBRT (32). Another prospective study showed WBRT and ASCT are both feasible and effective consolidation after high dose MTX based chemotherapy (33). The conditioning regimen is designed to allow the chemotherapeutic drugs to penetrate the CNS and thereby exert anti-tumor effects. One common conditioning regimen used for this particular scenario was primarily a combination of carmustine, etoposide (VP16), AraC, and melphalan (L-PAM) (BEAM regimen) or thiotepa-based treatments (34, 35). GOELAMS group reported an OS of 64% at 4 years in patients treated with BEAM regimen (34). Thiotepa-based and/or busulfan (BU)-based regimens have also demonstrated high efficacy (35–38). Busulfan and thiotepa (BuTT regimen) had an OS at 2 years of 48% (35). BuTT plus cyclophosphamide (TBC regimen) may improve the efficacy without neurotoxicity. Although TBC regimens demonstrated CR rates of >80%, they were associated with high treatment-related mortality, particularly in elderly patients (36, 38, 39). Among various other conditioning regimens, upfront L-PAM, cyclophosphamide, VP16, and dexamethasone (LEED) followed by ASCT was reported for patients with newly diagnosed PCNSL (40); no neurotoxicity was observed in the study. As a more intensive treatment strategy, combination of a high-dose regimen followed by ASCT and response-adapted WBRT was attempted, but WBRT increased the incidence of severe neurotoxicity (35, 41).

The prognosis of R/R PCNSL is poor. The median OS without treatment was reported to be only 2 months (42). Although there is no standard treatment strategy for R/R PCNSL, numerous studies have been reported. A retrospective analysis revealed that the overall response rates of high dose MTX rechallenge were 85% or higher (43, 44). However, this study included patients only who achieved CR after first line MTX-based therapies, and seven of 16 patients relapsed after the salvage therapy. High dose chemotherapy followed by ASCT is one of the available treatment strategies for R/R PCNSL for patients who are eligible for intensive treatment, but the 2-year overall survival probability was 45% (45, 46). Although these conventional chemotherapy-based approaches commonly showed high response rates, the duration of efficacy was insufficient. Target kinase inhibitors may be a tolerable approach for patients with R/R PCNSL. Ibrutinib, which is a first-in-class Bruton’s tyrosine kinase (BTK) inhibitor, showed 77% of clinical responses in patients with R/R PCNSL (47). Tirabrutinib, a second-generation BTK inhibitor, demonstrated an overall response rate of 64% in a phase 1/2 study (48). Although these therapies have shown varying degrees of efficacy with acceptable safety profiles, other treatment approaches, such as immunotherapy, are needed for R/R PCNSL to achieve a durable response. Rubenstein et al. conducted a phase I clinical trial of lenalidomide, a second-generation immunomodulatory agent, maintenance therapy in patients with PCNSL. They reported that the maintenance therapy with lenalidomide after rituximab plus lenalidomide therapy was feasible and prolonged the duration of response (49). This study served as a proof of concept for immunotherapy in PCNSL.

## CD19 CAR-T cell therapy in PCNSL

Pivotal clinical trials of CART19 demonstrated unprecedented efficacies in patients with R/R large B cell lymphoma or ALL, which led to the FDA approval of tisagenlecleucel (tisa-cel), axicabtagene ciloleucel (axi-cel), and lisocabtagene ciloleucel (liso-cel) (16, 50). Most recently, the FDA has approved axi-cel for the treatment of R/R follicular lymphoma and brexucabtagene autoleucel (brexu-cel) for R/R mantle cell lymphoma. Currently, various investigational drugs of CAR-T cell products are in the pipeline of clinical development. Clinical trials evaluating CAR-T cell therapy for large B cell lymphoma have mainly excluded patients with CNS lymphoma due to the risk of potential neurotoxicity with CAR-T cells, except for TRANSCEND NHL001 (22), which allowed secondary CNS involvement. According to Abramson et al., seven patients who enrolled in this study had secondary CNS lesions, and six of these patients were evaluable for CART19 efficacy. Three out of six patients (50%) achieved a CR, no patients had Grade 3-4 cytokine release syndrome (CRS), and two patients (33%) had Grade 3 neurological events. Some case studies have also evaluated the efficacy and safety of CART19 for secondary CNS lymphoma (15, 22–24, 51). Similar to the TRANSCEND study, these cases also demonstrated ORR of 30-50% with no cases of severe CRS and 0-30% of patients experiencing Grade 3-4 neurotoxicity. Based on the favorable effect of CART19 in secondary CNS lymphoma, CART19 cell therapy is becoming a potential treatment option for PCNSL. **Table 1** summarizes CART19 cell therapy approaches in PCNSL. Frigault et al. recently reported a prospective study of CART19 cell therapy mainly targeting PCNSL (52). This phase 1/2 study of tisa-cel in adults (median age 63 years, range 34-81 years) with R/R PCNSL enrolled a total of 13 patients and administered 12 patients with tisa-cel. One patient was excluded from the trial due to disease progression during CAR-T cell manufacturing. The majority of patients had the nongerminal center B-cell subtype of DLBCL. All patients had prior history of ibrutinib-based therapy, and patients were allowed to continue these therapies up to 3 months after CAR-T cell infusion. Patients received standard lymphodepletion chemotherapies of fludarabine (25 mg/m^2^/day) and cyclophosphamide (250 mg/m^2^/day) on days -5, -4, and -3 of CAR-T cell infusion. CAR-T cells were administered intravenously. Among 12 patients who received tisa-cel, seven patients responded to the treatment (ORR: 58.3%), which consisted of one partial response (PR) and six CRs (CR rate: 50%). Three patients have remained in remission within the follow-up period. CRS and immune cell-associated neurotoxicity syndrome (ICANS) were graded based on the American Society for Transplantation and Cellular Therapy (ASTCT) criteria (53) and occurred in seven (58%, Grade 1/2/3/4 = 7/0/0/0) and six patients (50%, Grade 1/2/3/4 = 3/2/1/0), respectively. No patient required tocilizumab to control CRS, and Grade 3 ICANS was reversible.

**Table 1:** Summary of CAR-T cell therapy in PCNSL.

Author	CAR-T cell product(s)	Co-stimulatory domains	Study Design	Number of patients	Median age (range)	Number of prior regimen (range)	Prior transplantation	Median follow-up period from infusion (range)	Response ORR/CR	Median duration of response (range)	CRS	ICANS	Ref (PMID)
Frigault, M. J.	Tisa-cel	4-1BB-CD3ζ	Phase 1/2	12	63 (34-81)	4 (2-6)	3	12.2 months (3.64-23.5)	58.3%/50%	N/A 3/7 patients have not progressed at data cut off	58.3% Grade 1/2/3/4=7/0/0/0 ASTCT criteria	50% Grade 1/2/3/4=3/2/1/0 ASTCT criteria	35167655
Alcantara M.	Tisa-cel Axi-cel	4-1BB-CD3ζ or CD28-CD3ζ	Retrospective	9 Tisa-cel: 7 Axi-cel: 2	67 (48-75)	3 (2-5)	7	8.5 months (1.2-15.3)	66.7%/55.6%	5.9 months (2.0-11.7) 4/6 patients have not progressed at data cut off	77.8% Grade 1/2/3/4=2/4/1/0 ASTCT criteria	55.6% Grade 1/2/3/4=2/1/1/1 ASTCT criteria	34871363
Siddiqi T.	CART19	CD28-CD3ζ	Retrospective	5	49 (42-53)	5 (2-12)	0	N/A	60%/60%	9.1 months (1.4-17.3)	42% Grade 1/2/3/4=3/2/0/0 Lee criteria	100% Grade 1/2/3/4=3/1/1/0 CTCAE 4.0	34492703
Li T.	CART19 CART22	4-1BB-CD28-CD3ζ	Prospective	1	49	3	0	N/A	PD	N/A	Grade 1 Lee Criteria	none CTCAE 5.0	32903866
Wu J.	CART19 CART22 followed by ASCT	4-1BB-CD3ζ	Prospective	4	44.5 (39-55)	3 (3-4)	0	14.2 months (1.4-24.2)	100%/75%	9.35 months (0.23-19.3)	75% Grade 1/2/3/4=3/0/0/0 ASTCT criteria	25% Grade 1/2/3/4=0/0/1/0 ASTCT criteria	34267187
Tu S.	CART19 CART70	CD28-CD27-CD3ζ	Case report	1	67	4	0	17 months	CR	17 months	none	none	31867275

CAR-T cell, chimeric antigen receptor-T cell; tisa-cel, tisagenlecleucel; axi-cel, axicabtagene ciloleuce; CART19, CD19 directed CAR-T cell; CART22, CD22 directed CAR-T cell; ASCT, autologous stem cell transplantation; CART70, CD70 directed CAR-T cell; ORR, overall response rate; CR, complete response; CRS, cytokine release syndrome; ICANS, immune effector cell-associated neurotoxicity syndrome; ASTCT, american society for transplantation and cellular therapy; CTCAE, common terminology criteria for adverse event.

The other two retrospective studies discussed in **Table 1** also suggested favorable outcomes of CART19 cell therapy in PCNSL. Alcantara et al. reported nine cases of R/R PCNSL treated with CART19 (seven cases of tisa-cel and two cases of axi-cel). Overall response at one month was observed in six of nine patients (67%), including CR in three of nine patients (30%). Median progression-free survival (PFS) was 122 days, and PFS increased to 210 days for responders. Toxicities were mild and manageable. Seven patients experienced CRS (77.8%, Grade 1/2/3/4 = 2/4/1/0), and five patients developed ICANS (55.6%, Grade 1/2/3/4 = 2/1/1/1). Siddiqi et al. also discussed a similar case cohort study (54) with five patients with PCNSL. Overall response was seen in three of five patients (60%), and all three of these patients achieved CR. It should be noted that patients were monitored for CRS and ICANS with Lee criteria (55) and Common Terminology Criteria for Adverse Events v4.0, respectively. CRS and ICANS were seen in all patients; the highest grade CRS was 2 (Grade 1/2/3/4 = 3/2/0/0), and the highest grade ICANS was 3 (Grade 1/2/3/4 = 3/1/1/0). All toxicities were reversible and tolerable, and there were no treatment-related deaths.

Although these are small studies, CART19 cell therapies in PCNSL were well tolerated and showed promising efficacy similar to non-CNS lymphomas. **Table 2** shows ongoing clinical trials of CAR-T cell therapy in patients with PCNSL.

**Table 2:** Clinical trials of CAR-T cell therapy in PCNSL.

Clinical Trial Identifier (ClinicalTrials.gov)	Title	Disease(s)	Population	Location	Study Design	Study Design	Interventions	Conditioning Regimen
NCT04443829	Immunotherapy Using CAR T-cells to Target CD19 for Relapsed/Refractory CD19+ PCNSL Lymphoma (CAROUSEL)	R/R PCNSL	>16 years	University College, London	Phase 1	Single-center, non-randomised, open label	Dose 1:CART19 250 x 106 cells iv At Day 28 response SD or PD →Dose 2: 25 x 106 cells icv	Cy 60mg/kg on Day -6 Flu 30mg/m2 on Day -5 to Day -3 Pembrolizumab 200mg on Day -1
NCT04134117	Pilot Study of Tisagenlecleucel, CD19-targeted CAR T Cells, in Patients With PCNSL	R/R PCNSL	>18 years	Massachusetts General Hospital	Phase 1	Single-center, non-randomised, open label	One time single predetermined dose level of tisa-cel will be infused intravenously	Flu/Cy
NCT04608487	A Phase I Study of Anti-CD19 CAR T-cell Therapy With Axi-cel in Patients With Relapsed/Refractory Primary and Secondary CNS Lymphoma	Cohort 1: R/R PCNSL, secondary CNSL without DLBCL, HGBL, PMBL, tFL Cohort 2: R/R systemic DLBCL, HGBL, PMBL, tFL with either active CNSL or previously treated CNSL	>18 years	Dana Farber Cancer Institute	Phase 1	Single-center, non-randomised, open label	One time single predetermined dose level axi-cel will be infused intravenously	Flu/Cy
NCT04464200	A Phase I Study of CD19-Targeted 19(T2)28z1xx CAR Modified T Cells in Adult Patients With Relapsed or Refractory B-cell Malignancies	DLBCL, PMBL, tFL, CLL, indolent NHL, MZL, Waldenstrom Macroglobulinemia, Burkitt lymphoma, PCNSL	>18 years	Memorial Sloan Kettering Cancer Center	Phase 1 Dose escalation study	Single-center, non-randomised, open label	CD19-Targeted 19(T2)28z1xx CAR T cells will be infused intravenously	Flu/Cy

CAR-T cell, chimeric antigen receptor-T cell; tisa-cel, tisagenlecleucel; axi-cel, axicabtagene ciloleuce; CART19, CD19 directed CAR-T cell; R/R PCNSL, relapsed refractory primary central nervous system lymphoma; DLBCL, diffuse large B cell lymphoma; HGBL, high-grade B cell lymphoma; PMBL, primary mediastinal B cell lymphoma; tFL, transformed follicular lymphoma; NHL, non-Hodgkin lymphoma, MZL, marginal zone lymphoma; SD, stable disease; PD, progressive disease; iv, intravenous injection, icv, Intracerebroventricular injection; Cy, cytarabine; Flu, fludarabine.

## Toxicities of CAR-T cell therapy in PCNSL

According to the ASTCT, CRS is characterized by fever, hypotension, hypoxia, and end organ dysfunction (53). CRS is associated with massive expansion of infused cells *in vivo* as well as extreme elevation of multiple cytokines/chemokines. CRS develops in 50-100% of cases after CART19 cell therapy (15, 56, 57) and is significantly correlated with higher disease burden at baseline and *in vivo* CAR-T cell proliferation (58–61). ICANS is the second-most noted life-threatening adverse event associated with CAR-T cell therapy and is characterized by generalized cerebral edema, confusion, obtundation, aphasia, motor weakness, and occasionally, seizures (53, 62). Any grade of ICANS occurs in up to 70% of patients, and Grade 3-4 is reported in 20-30% of patients (15, 16, 56, 60). Overall, CRS and ICANS are common and can be fatal.

While the exact mechanisms of ICANS remain unknown, Parker et al. have recently shown that pericyte populations in the brain express CD19, pointing to one potential mechanism of ICANS (63). The incidences of ICANS also reported in patients treated with CD19-targeting bispecific antibodies (64). However, cases of ICANS have been reported with CD22 (65) and BCMA (66) directed CAR-T cell therapy, which makes difficult to conclude that ICANS occurrence is owing to the presence of the target antigen within the CNS.

Historically, patients with PCNSL or secondary CNS lymphoma were excluded from most pivotal CAR-T cell therapy trials due to concerns about the increased risk of ICANS. A recent CART19 clinical study in PCNSL reported by Frigault et al. (52) and two retrospective analyses from Alcantara et al. (25) and Siddiqi et al. (54) showed that patients with PCNSL treated with CART19 cell therapy developed reversible and tolerable ICANS.

## Local administration of CAR-T Cells

Most clinical trials of CAR-T cell therapy in hematological malignancies as well as solid tumors have been conducted using intravenous administration (67). However, limited efficacies were reported in cases with bulky diseases, PCNSL, or solid tumors due to the poor trafficking of CAR-T cells to the tumor site (68). However, there is increasing evidence to suggest that local application of CAR-T cells may increase tumor penetration and efficacy in some cases.

Local administration of CAR T-cells was recently tested in an immunocompromised NOD-SCID-γ^-^/^-^ mouse model of PCNSL (69). The researchers established an orthotopic PCNSL mouse model by intracranial injection of human CD19^+^ lymphoma cell lines (Daudi or JeKo-1). The CART19 cells (CD28ζ co-stimulated) were administered through a single infusion, either intracerebroventricular or intravenous. Interestingly, bioluminescence imaging revealed intracerebroventricularly delivered CAR-T cells were able to completely and durably eradicate both CNS and systemic lymphoma. On the other hand, CAR-T cells delivered through intravenous injection failed to show anti-tumor effects. They also showed that intracerebroventricularly infused CAR-T cells exhibited similar trafficking but significantly better proliferation and persistence compared to intravenously infused CAR-T cells. Interestingly, intracerebroventricularly delivered CAR-T cells exhibited a higher percentage of memory phenotype than intravenously administered CAR-T cells. The authors concluded that exposure of CAR-T cells to CSF leads to a metabolic reprogramming that favors the formation of memory T cells, as inhibition of glycolysis enhances memory T cell phenotypes (70, 71).

Locally administered CAR-T cells in PCNSL are now being tested in a phase I clinical trial. All patients will be treated first with CAR T-cells intravenously in this study. If patients do not show response (stable disease (SD) or progressive disease (PD) at day 28) to the first round of CAR-T cell infusion and in the absence of severe CART-related toxicity, patients will be potentially eligible for a second round of CAR-T cells administered intracerebroventricularly *via* an Ommaya reservoir (**Table 2**) (NCT04443829).

Similar to local CART19 injection in the PCNSL mouse model by Wang et al. (69), B7H3-targeted CAR-T cell therapy was tested in orthotopic atypical teratoid/rhabdoid tumor xenografts with either intracerebroventricular or intravenous injection. Theruvath et al. demonstrated that intracerebroventricular administration of B7H3 CAR-T cells resulted in significantly better overall survival and anti-tumor effects, with faster CAR-T expansion *in vivo* and reduced systemic inflammatory cytokines compared to intravenous injection (72).

These recently reported preclinical data provide rationale to further assess local administration of CAR-T cell to treat PCNSL.

## Targeting multiple antigens with CAR-T cells

To prevent tumor relapse after CAR-T cell therapy due to loss of the target antigen, there have been efforts to establish CAR-T cell strategies to recognize multiple tumor antigens in preclinical models (73–78), and some dual-targeted CAR-T products have already been tested in clinical trials in patients with systemic lymphoma or multiple myeloma (79–84). A similar concept is also being applied to PCNSL. Wu et al. have conducted a clinical trial of sequential therapy with ASCT followed by anti-CD19 and CD22 cocktail CAR-T cell therapy. Thirteen patients with CNS lymphoma were enrolled in this study (four PCNSL and nine secondary CNS lymphoma). Two patients (one PCNSL and one secondary CNS lymphoma) achieved CR at the time of CAR-T cell infusion and maintained durable remission. Overall response was observed in nine of 11 patients (82%, three PCNSL and six secondary CNS lymphoma), including CR in six of 11 patients (55%, two PCNSL and four secondary CNS lymphoma). CRS and ICANS occurred in 11 patients (85%, Grade 1/2/3/4 = 9/2/0/0) and three patients (23%, Grade 1/2/3/4 = 2/0/1/0), respectively. All adverse events were reversible and tolerable, and there were no deaths related to the treatment (85).

Li et al. also reported five patients with CNS lymphoma (one patient with PCNSL and four patients with secondary CNS lymphoma) who underwent anti-CD19 and CD22 cocktail CAR-T cell therapy with a follow-up of 6-16 months. Two patients achieved CR, and three other patients achieved PR. However, four patients developed relapse within 3 to 8 months after CAR-T cell therapy. Unlike the study from Wu e al., this trial did not perform ASCT prior to CAR-T cell infusion. Therefore, the baseline tumor burden at the time of CAR-T cell administration was higher. Furthermore, the authors mentioned that the early relapse was seen due to the immunosuppressive tumor microenvironment (TME) of the CNS, which was unrelated to antigen escape (86). Tu et al. reported a case report of dual-targeting CD19/CD70 CAR-T cells in a patient with R/R PCNSL. Durable remission at 17 months was observed after the treatment (**Table 1**) (87).

Given these results, dual targeting of different antigens on tumor cells may not contribute to favorable outcomes. To achieve long-term durable response, it may be crucial to decrease the tumor burden before CAR-T cell treatment. Moreover, targeting not only the tumor cells but also the TME may enhance anti-tumor activity of CAR-T cells and prevent an early relapse after CAR-T cell treatment.

## Combination with other immunotherapies

The contribution of the TME to tumor growth and therapy resistance has been recognized in most malignancies and also applies to PCNSL. In the last decade, the field of oncology has been transformed by immunotherapies, including antibodies directed against immune checkpoints or ligands, such as PD-1/PD-L1 or CTLA-4 (88, 89). Many studies have demonstrated that the presence of PD-1^+^ tumor-infiltrating lymphocytes and PD-L1^+^ microglial cells, tumor-associated macrophages, and tumor cells within the TME correlate with patient outcomes. Specifically, Chapuy et al. reported PCNSL and primary testicular lymphoma (PTL) showed a higher 9p24 amplification compared to systemic DLBCL. A 9p24 amplification in malignant lymphoma correlates with PD-L1/PD-L2 deregulation and results in increased PD-L1 expression on tumor cells (90–92). Takashima et al. performed next generation sequencing on PCNSL samples and discovered that high expression of LAG3, PD-1, and PD-L2 were associated with poor prognosis (93). PD-1 blocking therapy in preclinical PCNSL models as well as early clinical data indicate its efficacy in PCNSL. Qiu et al. created a mouse PCNSL model by injecting the murine B-cell lymphoma cell line, A20, to the periventricular area. PD-1 antibody treatment resulted in prolonged overall survival, increased CD8^+^ tumor-infiltrating lymphocytes, and complete eradication of tumor cells (94). Nayak et al. treated four patients with PCNSL and one patient with CNS relapse of PTL with the PD-1 blocking antibody, nivolumab. Overall response was 100%, including CR rates of 80% (three patients with PCNSL and one patient with CNS relapse of PTL). One patient developed Grade 2 pruritus, and another patient experienced Grade 2 fatigue. The authors concluded that these are nivolumab-related toxicities. However, one patient developed worsening of baseline renal functions (Grade 4) and required hemodialysis. Renal biopsy showed no evidence of interstitial nephritis, so this event was considered to be unrelated to nivolumab treatment (95). Another study reported a case with R/R PCNSL who was successfully treated with nivolumab and dendritic cell vaccination (96).

These checkpoint inhibitors demonstrate anti-tumor effects *via* activated T lymphocytes; therefore, combination therapy of PD-1 blocking antibody and CAR-T cell therapy may increase CAR-T cell anti-tumor efficacy in the treatment of PCNSL. Based on these preclinical and clinical data, the combination of CAR-T cell therapy with the PD-1 inhibitor, pembrolizumab, to overcome the negative effects of immunosuppressive cells in the TME is being evaluated in patients with R/R PCNSL (**Table 2**) (CAROUSEL Trial, phase I clinical trial, NCT04443829).

## Can CAR-T cell therapy be applied to human immunodeficiency virus (HIV)-1-related PCNSL?

Patients with HIV-1 are at increased risk for PCNSL compared to uninfected populations. From a retrospective study, among all patients with PCNSL, 19% had HIV-1 (97). As we discussed earlier, CAR-T cell therapy may be a feasible treatment option for patients with PCNSL. Since pivotal clinical trials of CAR-T cell therapy excluded patients who were positive for HIV-1, it is difficult to assess whether CAR-T cell therapy is safe and efficacious in patients with HIV-1-positive PCNSL. However, the use of CAR-T cell therapy in HIV-1-positive PCNSL is increasing. Exclusion criteria for CAR-T cell products approved by the FDA or other regulatory agencies do not include HIV-1 positivity. In fact, two HIV-1-infected patients with high-grade B-cell lymphoma were successfully treated with CART19 cell therapy (98). In this report, CAR-T cells were successfully manufactured in HIV-infected patients who were receiving antiretroviral therapy. CAR-T cell products were administered safely and led patients into remission. One of the original concepts of CAR-T cell therapy was to target HIV-1-infected T cells with anti-HIV CAR-T cells (99). In 2002, Deeks et al. reported CD4ζ-modified first-generation anti-HIV CAR-T cells in a phase II randomized study (100). Although no therapeutic efficacy was demonstrated, anti-HIV-1 CAR-T cells were successfully generated from HIV-1-infected patients, and long-term engraftment was reported. More recently, anti-HIV-1 CAR-T cell technology was reported by Liu et al. They developed HIV-1 broadly neutralizing antibody (bNAb)-derived CAR-T cells, which contain both CD28 and 4-1BB intracellular costimulatory domains, and administered them to 14 patients with HIV-1. These CAR-T cells significantly reduced cell-associated viral RNA and intact proviruses (101). Additionally, an *in vivo* study reported that bNAb-derived CAR-T cells could enhance the efficacy of PD-1 blockade (102). These results encourage the application of CART19 cell therapy in HIV-1-positive PCNSL. Novel and promising strategies can further improve efficacy and favorable outcomes. For example, dual targeting CD19 along with the membrane-proximal external region, which is a highly conserved region of the envelope glycoprotein gp41 subunit near the viral envelope (103–105), with CAR-T cells or combination of CAR-T cell cocktails targeting both CD19^+^ and HIV-infected cells might synergetically enhance the efficacy. 

## Discussion

As we discussed in this review article, the standard therapy for PCNSL has not been established, and numerous problems and obstacles must be overcome to induce durable remission. CAR-T cell therapy may be a promising solution to advance the treatment approach for PCNSL. Historically, radiation therapy, high-dose MTX or AraC, and consolidation therapy with autologous transplantation have been applied for the treatment of PCNSL due to the ability of these therapies to penetrate the blood-brain barrier. However, serious adverse events including leukoencephalopathy or neurotoxicity are common. Unlike these treatments, CAR-T cell therapies in patients with PCNSL has rarely resulted in cases of leukoencephalopathy. In terms of neurotoxicity, most cases of PCNSL treated with CAR-T cell therapy demonstrated that treatment-related toxicities were reversible and tolerable. Cook et al. have recently reported a meta-analysis of CAR-T cell therapy in patients with PCNSL or secondary CNSL. Similar to the studies that we described in this review article that toxicities were comparable to that of systemic large B cell lymphoma with no increased incidence of neurotoxicity. They also described that encouraging efficacy of CAR-T cell therapy were demonstrated with PCNSL and secondary CNSL (106).

The major drawback of CAR-T cell therapy in PCNSL is low durable response, similar to systemic lymphoma or leukemia. The lack of durable responses has been widely attributed to the immunosuppressive TME and resultant T cell dysfunction. Lack of trafficking of CAR-T cells to the tumor site also correlates with the low durability of CAR-T cells in PCNSL. Novel combination therapies with other agents, including kinase inhibitors or checkpoint inhibitors, or local administration of CAR-T cell therapy are currently being investigated in clinical trials. CAR-T cell therapy in PCNSL has potential to change overall treatment strategy. For example, the combination of CAR-T cell therapy with conventional induction therapy with high-dose MTX may improve the response rate and durable response as a first line therapy for PCNSL. This would reduce patients’ exposure to high-dose chemotherapies and thereby reduce the risk of associated leukoencephalopathy or neurotoxicity. Overall, emerging data discussed in this manuscript encourage further investigation of the use of CART19 cell therapy for the treatment of PCNSL.

## Author contributions

KM, HY, and RS wrote the manuscript. All authors contributed to the article and approved the submitted version.

## References

[1] O'Neill BP Decker PA Tieu C Cerhan JR. The changing incidence of primary central nervous system lymphoma is driven primarily by the changing incidence in young and middle-aged men and differs from time trends in systemic diffuse large B -cell non-Hodgkin's lymphoma. Am J Hematol (2013) 88:997–1000. doi: 10.1002/ajh.23551 23873804 PMC4020348

[2] Shiels MS Pfeiffer RM Besson C Clarke CA Morton LM Nogueira L. Trends in primary central nervous system lymphoma incidence and survival in the U.S. Brit J Haematol (2016) 174:417–24. doi: 10.1111/bjh.14073 PMC4961566 27018254

[3] Grommes C DeAngelis LM. Primary CNS lymphoma. J Clin Oncol (2017) 35:2410–8. doi: 10.1200/JCO.2017.72.7602 PMC5516483 28640701

[4] Abrey LE Batchelor TT Ferreri AJ Gospodarowicz M Pulczynski EJ Zucca E. Report of an international workshop to standardize baseline evaluation and response criteria for primary CNS lymphoma. J Clin Oncol (2005) 23:5034–43. doi: 10.1200/JCO.2005.13.524 15955902

[5] Louis DN Perry A Reifenberger G von Deimling A Figarella-Branger D Cavenee WK. The 2016 world health organization classification of tumors of the central nervous system: a summary. Acta Neuropathol (2016) 131:803–20. doi: 10.1007/s00401-016-1545-1 27157931

[6] Han CH Batchelor TT. Diagnosis and management of primary central nervous system lymphoma. Cancer (2017) 123:4314–24. doi: 10.1002/cncr.30965 28950405

[7] Grupp SA Kalos M Barrett D Aplenc R Porter DL Rheingold SR. Chimeric antigen receptor-modified T cells for acute lymphoid leukemia. N Engl J Med (2013) 368:1509–18. doi: 10.1056/NEJMoa1215134 PMC4058440 23527958

[8] Porter DL Hwang WT Frey NV Lacey SF Shaw PA Loren AW. Chimeric antigen receptor T cells persist and induce sustained remissions in relapsed refractory chronic lymphocytic leukemia. Sci Transl Med (2015) 7(303): ra139. doi: 10.1126/scitranslmed.aac5415 PMC5909068 26333935

[9] Kenderian SS Porter DL Gill S. Chimeric antigen receptor T cells and hematopoietic cell transplantation: How not to put the CART before the horse. Biol Blood Marrow Transplant (2017) 23:235–46. doi: 10.1016/j.bbmt.2016.09.002 PMC5237606 27638367

[10] Bouchkouj N Kasamon YL de Claro RA George B Lin X Lee S. FDA Approval summary: Axicabtagene ciloleucel for relapsed or refractory Large b-cell lymphoma. Clin Cancer Res (2019) 25:1702–8. doi: 10.1158/1078-0432.CCR-18-2743 30413526

[11] Chow VA Shadman M Gopal AK. Translating anti-CD19 CAR T-cell therapy into clinical practice for relapsed/refractory diffuse large b-cell lymphoma. Blood (2018) 132:777–81. doi: 10.1182/blood-2018-04-839217 PMC6107879 29914976

[12] Locke FL Neelapu SS Bartlett NL Siddiqi T Chavez JC Hosing CM. Phase 1 results of ZUMA-1: A multicenter study of KTE-C19 anti-CD19 CAR T cell therapy in refractory aggressive lymphoma. Mol Ther (2017) 25:285–95. doi: 10.1016/j.ymthe.2016.10.020 PMC5363293 28129122

[13] Better M Chiruvolu V Oliver J Lowe E Rossi JM Perez A. Production of KTE-C19 (Anti-CD19 CAR T cells) for ZUMA-1: A phase 1/2 multi-center study evaluating safety and efficacy in subjects with refractory aggressive non-Hodgkin lymphoma (NHL). Mol Ther (2016) 24:S115–5. doi: 10.1016/S1525-0016(16)33096-9

[14] Neelapu SS Locke FL Bartlett NL Lekakis LJ Miklos DB Jacobsen ED. Long-term follow-up ZUMA-1: A pivotal trial of axicabtagene ciloleucel (Axi-cel; KTE-C19) in patients with refractory aggressive non-Hodgkin lymphoma (NHL). Blood 130 (2017). doi: 10.1002/hon.2437_7

[15] Neelapu SS Locke FL Bartlett NL Lekakis LJ Miklos DB Jacobson CA. Axicabtagene ciloleucel CAR T-cell therapy in refractory Large b-cell lymphoma. N Engl J Med (2017) 377:2531–44. doi: 10.1056/NEJMoa1707447 PMC5882485 29226797

[16] Schuster SJ Bishop MR Tam CS Waller EK Borchmann P McGuirk JP. Tisagenlecleucel in adult relapsed or refractory diffuse Large b-cell lymphoma. N Engl J Med (2019) 380:45–56. doi: 10.1056/NEJMoa1804980 30501490

[17] Wang M Munoz J Goy A Locke FL Jacobson CA Hill BT. KTE-X19 CAR T-cell therapy in relapsed or refractory mantle-cell lymphoma. N Engl J Med (2020) 382:1331–42. doi: 10.1056/NEJMoa1914347 PMC7731441 32242358

[18] Jacobson C Chavez JC Sehgal AR William BM Munoz J Salles G. Primary analysis of zuma-5: A phase 2 study of axicabtagene ciloleucel (Axi-cel) in patients with Relapsed/Refractory (R/R) indolent non-Hodgkin lymphoma (iNHL). Blood (2020) 136:40–1. doi: 10.1182/blood-2020-136834

[19] Munshi NC Anderson LD Shah N Madduri D Berdeja J Lonial S. Idecabtagene vicleucel in relapsed and refractory multiple myeloma. New Engl J Med (2021) 384:705–16. doi: 10.1056/NEJMoa2024850 33626253

[20] Locke FL Miklos DB Jacobson CA Perales M-A Kersten M-J Oluwole OO. Axicabtagene ciloleucel as second-line therapy for Large b-cell lymphoma. New Engl J Med (2021) 386:640–54. doi: 10.1056/NEJMoa2116133 34891224

[21] Bishop MR Dickinson M Purtill D Barba P Santoro A Hamad N. Second-line tisagenlecleucel or standard care in aggressive b-cell lymphoma. New Engl J Med (2021) 386:629–39. doi: 10.1056/NEJMoa2116596 34904798

[22] Abramson JS Palomba ML Gordon LI Lunning MA Wang M Arnason J. Lisocabtagene maraleucel for patients with relapsed or refractory large b-cell lymphomas (TRANSCEND NHL 001): a multicentre seamless design study. Lancet (2020) 396:839–52. doi: 10.1016/S0140-6736(20)31366-0 32888407

[23] Frigault MJ Dietrich J Martinez-Lage M Leick M Choi BD DeFilipp Z. Tisagenlecleucel CAR T-cell therapy in secondary CNS lymphoma. Blood (2019) 134:860–6. doi: 10.1182/blood.2019001694 PMC7022436 31320380

[24] Abramson JS McGree B Noyes S Plummer S Wong C Chen YB. Anti-CD19 CAR T cells in CNS diffuse Large-B-Cell lymphoma. N Engl J Med (2017) 377:783–4. doi: 10.1056/NEJMc1704610 28834486

[25] Alcantara M Houillier C Blonski M Rubio MT Willems L Rascalou AW. CAR T-cell therapy in primary central nervous system lymphoma: the clinical experience of the French LOC network. Blood (2022) 139:792–6. doi: 10.1182/blood.2021012932 PMC8814680 34871363

[26] Brandsma D Bromberg JEC. Primary CNS lymphoma in HIV infection. Handb Clin Neurol (2018) 152:177–86. doi: 10.1016/B978-0-444-63849-6.00014-1 29604975

[27] Nelson DF Martz KL Bonner H Nelson JS Newall J Kerman HD. Non-hodgkin's lymphoma of the brain: can high dose, large volume radiation therapy improve survival? report on a prospective trial by the radiation therapy oncology group (RTOG): RTOG 8315. Int J Radiat Oncol Biol Phys (1992) 23:9–17. doi: 10.1016/0360-3016(92)90538-S 1572835

[28] Rubenstein JL Hsi ED Johnson JL Jung SH Nakashima MO Grant B. Intensive chemotherapy and immunotherapy in patients with newly diagnosed primary CNS lymphoma: CALGB 50202 (Alliance 50202). J Clin Oncol (2013) 31(25). doi: 10.1200/JCO.2012.46.9957 PMC3753699 23569323

[29] Omuro A Chinot O Taillandier L Ghesquieres H Soussain C Delwail V. Methotrexate and temozolomide versus methotrexate, procarbazine, vincristine, and cytarabine for primary CNS lymphoma in an elderly population: an intergroup ANOCEF-GOELAMS randomised phase 2 trial. Lancet Haematol (2015) 2:e251–9. doi: 10.1016/S2352-3026(15)00074-5 26688235

[30] Shah GD Yahalom J Correa DD Lai RK Raizer JJ Schiff D. Combined immunochemotherapy with reduced whole-brain radiotherapy for newly diagnosed primary CNS lymphoma. J Clin Oncol (2007) 25:4730–5. doi: 10.1200/JCO.2007.12.5062 17947720

[31] Thiel E Korfel A Martus P Kanz L Griesinger F Rauch M. High-dose methotrexate with or without whole brain radiotherapy for primary CNS lymphoma (G-PCNSL-SG-1): a phase 3, randomised, non-inferiority trial. Lancet Oncol (2010) 11:1036–47. doi: 10.1016/S1470-2045(10)70229-1 20970380

[32] Houillier C Taillandier L Dureau S Lamy T Laadhari M Chinot O. Radiotherapy or autologous stem-cell transplantation for primary CNS lymphoma in patients 60 years of age and younger: Results of the intergroup ANOCEF-GOELAMS randomized phase II PRECIS study. J Clin Oncol (2019) 37:823–33. doi: 10.1200/JCO.18.00306 30785830

[33] Ferreri AJM Cwynarski K Pulczynski E Fox CP Schorb E La Rosée P. Whole-brain radiotherapy or autologous stem-cell transplantation as consolidation strategies after high-dose methotrexate-based chemoimmunotherapy in patients with primary CNS lymphoma: results of the second randomisation of the international extranodal lymphoma study group-32 phase 2 trial. Lancet Haematol (2017) 4:e510–23. doi: 10.1016/S2352-3026(17)30174-6 29054815

[34] Colombat P Lemevel A Bertrand P Delwail V Rachieru P Brion A. High-dose chemotherapy with autologous stem cell transplantation as first-line therapy for primary CNS lymphoma in patients younger than 60 years: a multicenter phase II study of the GOELAMS group. Bone Marrow Transplant (2006) 38:417–20. doi: 10.1038/sj.bmt.1705452 16951691

[35] Montemurro M Kiefer T Schüler F Al-Ali HK Wolf HH Herbst R. Primary central nervous system lymphoma treated with high-dose methotrexate, high-dose busulfan/thiotepa, autologous stem-cell transplantation and response-adapted whole-brain radiotherapy: results of the multicenter ostdeutsche studiengruppe hamato-onkologie OSHO-53 phase II study. Ann Oncol (2007) 18:665–71. doi: 10.1093/annonc/mdl458 17185743

[36] Cheng T Forsyth P Chaudhry A Morris D Glück S Russell JA. Busulfan, cyclophosphamide and ASCT without whole-brain radiotherapy for poor prognosis primary CNS lymphoma. Bone Marrow Transplant (2003) 31:679–85. doi: 10.1038/sj.bmt.1703917 12692608

[37] Cote GM Hochberg EP Muzikansky A Hochberg FH Drappatz J McAfee SL. Autologous stem cell transplantation with thiotepa, busulfan, and cyclophosphamide (TBC) conditioning in patients with CNS involvement by non-Hodgkin lymphoma. Biol Blood Marrow Transplant (2012) 18:76–83. doi: 10.1016/j.bbmt.2011.07.006 21749848

[38] Omuro A Correa DD DeAngelis LM Moskowitz CH Matasar MJ Kaley TJ. R-MPV followed by high-dose chemotherapy with TBC and autologous stem-cell transplant for newly diagnosed primary CNS lymphoma. Blood (2015) 125:1403–10. doi: 10.1182/blood-2014-10-604561 PMC4342354 25568347

[39] Alimohamed N Daly A Owen C Duggan P Stewart DA thiotepa U. Busulfan, cyclophosphamide, and autologous stem cell transplantation for primary CNS lymphoma: a single centre experience. Leuk Lymphoma (2012) 53:862–7. doi: 10.3109/10428194.2011.633250 22023529

[40] Miyao K Sakemura R Imai K Sakai T Tsushita N Kato T. Upfront autologous stem-cell transplantation with melphalan, cyclophosphamide, etoposide, and dexamethasone (LEED) in patients with newly diagnosed primary central nervous system lymphoma. Int J Hematol (2014) 100:152–8. doi: 10.1007/s12185-014-1608-9 24947495

[41] Kiefer T Hirt C Späth C Schüler F Al-Ali HK Wolf HH. Long-term follow-up of high-dose chemotherapy with autologous stem-cell transplantation and response-adapted whole-brain radiotherapy for newly diagnosed primary CNS lymphoma: results of the multicenter ostdeutsche studiengruppe hamatologie und onkologie OSHO-53 phase II study. Ann Oncol (2012) 23:1809–12. doi: 10.1093/annonc/mdr553 22115927

[42] Reni M Ferreri AJ Villa E. Second-line treatment for primary central nervous system lymphoma. Br J Cancer (1999) 79:530–4. doi: 10.1038/sj.bjc.6690083 PMC2362452 10027325

[43] Plotkin SR Betensky RA Hochberg FH Grossman SA Lesser GJ Nabors LB. Treatment of relapsed central nervous system lymphoma with high-dose methotrexate. Clin Cancer Res (2004) 10:5643–6. doi: 10.1158/1078-0432.CCR-04-0159 15355887

[44] Pentsova E Deangelis LM Omuro A. Methotrexate re-challenge for recurrent primary central nervous system lymphoma. J Neurooncol (2014) 117:161–5. doi: 10.1007/s11060-014-1370-0 PMC5256683 24481997

[45] Welch MR Sauter CS Matasar MJ Faivre G Weaver SA Moskowitz CH. Autologous stem cell transplant in recurrent or refractory primary or secondary central nervous system lymphoma using thiotepa, busulfan and cyclophosphamide. Leuk Lymphoma (2015) 56:361–7. doi: 10.3109/10428194.2014.916800 24745937

[46] Soussain C Hoang-Xuan K Taillandier L Fourme E Choquet S Witz F. Intensive chemotherapy followed by hematopoietic stem-cell rescue for refractory and recurrent primary CNS and intraocular lymphoma: Société française de greffe de moëlle osseuse-thérapie cellulaire. J Clin Oncol (2008) 26:2512–8. doi: 10.1200/JCO.2007.13.5533 18413641

[47] Grommes C Pastore A Palaskas N Tang SS Campos C Schartz D. Ibrutinib unmasks critical role of bruton tyrosine kinase in primary CNS lymphoma. Cancer Discov (2017) 7:1018–29. doi: 10.1158/2159-8290.CD-17-0613 PMC5581705 28619981

[48] Narita Y Nagane M Mishima K Terui Y Arakawa Y Yonezawa H. Phase I/II study of tirabrutinib, a second-generation bruton's tyrosine kinase inhibitor, in relapsed/refractory primary central nervous system lymphoma. Neuro Oncol (2021) 23:122–33. doi: 10.1093/neuonc/noaa145 PMC7850159 32583848

[49] Rubenstein JL Geng H Fraser EJ Formaker P Chen L Sharma J. Phase 1 investigation of lenalidomide/rituximab plus outcomes of lenalidomide maintenance in relapsed CNS lymphoma. Blood Adv (2018) 2:1595–607. doi: 10.1182/bloodadvances.2017014845 PMC6039666 29986852

[50] Locke FL Ghobadi A Jacobson CA Miklos DB Lekakis LJ Oluwole OO. Long-term safety and activity of axicabtagene ciloleucel in refractory large b-cell lymphoma (ZUMA-1): a single-arm, multicentre, phase 1-2 trial. Lancet Oncol (2019) 20:31–42. doi: 10.1016/S1470-2045(18)30864-7 30518502 PMC6733402

[51] Karschnia P Rejeski K Winkelmann M Schöberl F Bücklein VL Blumenberg V. Toxicities and response rates of secondary CNS lymphoma after adoptive immunotherapy with CD19-directed chimeric antigen receptor T cells. Neurology (2022) 98:884–9. doi: 10.1212/WNL.0000000000200608 PMC9169944 35351785

[52] Frigault MJ Dietrich J Gallagher K Roschewski M Jordan JT Forst D. Safety and efficacy of tisagenlecleucel in primary CNS lymphoma: a phase 1/2 clinical trial. Blood (2022) 139:2306–15. doi: 10.1182/blood.2021014738 PMC9012129 35167655

[53] Lee DW Santomasso BD Locke FL Ghobadi A Turtle CJ Brudno JN. ASTCT consensus grading for cytokine release syndrome and neurologic toxicity associated with immune effector cells. Biol Blood Marrow Transplant (2019) 25:625–38. doi: 10.1016/j.bbmt.2018.12.758 PMC12180426 30592986

[54] Siddiqi T Wang X Blanchard MS Wagner JR Popplewell LL Budde LE. CD19-directed CAR T-cell therapy for treatment of primary CNS lymphoma. Blood Adv (2021) 5:4059–63. doi: 10.1182/bloodadvances.2020004106 PMC8945630 34492703

[55] Lee DW Gardner R Porter DL Louis CU Ahmed N Jensen M. Current concepts in the diagnosis and management of cytokine release syndrome. Blood (2014) 124:188–95. doi: 10.1182/blood-2014-05-552729 PMC4093680 24876563

[56] Maude SL Laetsch TW Buechner J Rives S Boyer M Bittencourt H. Tisagenlecleucel in children and young adults with b-cell lymphoblastic leukemia. N Engl J Med (2018) 378:439–48. doi: 10.1056/NEJMoa1709866 PMC5996391 29385370

[57] Gust J Hay KA Hanafi LA Li D Myerson D Gonzalez-Cuyar LF. Endothelial activation and blood-brain barrier disruption in neurotoxicity after adoptive immunotherapy with CD19 CAR-T cells. Cancer Discovery (2017) 7:1404–19. doi: 10.1158/2159-8290.CD-17-0698 PMC5718945 29025771

[58] Lee DW Kochenderfer JN Stetler-Stevenson M Cui YK Delbrook C Feldman SA. T Cells expressing CD19 chimeric antigen receptors for acute lymphoblastic leukaemia in children and young adults: a phase 1 dose-escalation trial. Lancet (2015) 385:517–28. doi: 10.1016/S0140-6736(14)61403-3 PMC7065359 25319501

[59] Maude SL Frey N Shaw PA Aplenc R Barrett DM Bunin NJ. Chimeric antigen receptor T cells for sustained remissions in leukemia. N Engl J Med (2014) 371:1507–17. doi: 10.1056/NEJMoa1407222 PMC4267531 25317870

[60] Park JH Riviere I Gonen M Wang X Senechal B Curran KJ. Long-term follow-up of CD19 CAR therapy in acute lymphoblastic leukemia. N Engl J Med (2018) 378:449–59. doi: 10.1056/NEJMoa1709919 PMC6637939 29385376

[61] Turtle CJ Hanafi LA Berger C Gooley TA Cherian S Hudecek M. CD19 CAR-T cells of defined CD4+:CD8+ composition in adult b cell ALL patients. J Clin Invest (2016) 126:2123–38. doi: 10.1172/JCI85309 PMC4887159 27111235

[62] Hunter BD Jacobson CA. CAR T-cell associated neurotoxicity: Mechanisms, clinicopathologic correlates, and future directions. J Natl Cancer Inst (2019) 111:646–54. doi: 10.1093/jnci/djz017 30753567

[63] Parker KR Migliorini D Perkey E Yost KE Bhaduri A Bagga P. Single-cell analyses identify brain mural cells expressing CD19 as potential off-tumor targets for CAR-T immunotherapies. Cell (2020) 183:126–142.e17. doi: 10.1016/j.cell.2020.08.022 32961131 PMC7640763

[64] Subklewe M. BiTEs better than CAR T cells. Blood Adv (2021) 5:607–12. doi: 10.1182/bloodadvances.2020001792 PMC7839370 33496755

[65] Fry TJ Shah NN Orentas RJ Stetler-Stevenson M Yuan CM Ramakrishna S. CD22-targeted CAR T cells induce remission in b-ALL that is naive or resistant to CD19-targeted CAR immunotherapy. Nat Med (2018) 24:20–8. doi: 10.1038/nm.4441 PMC5774642 29155426

[66] Garfall AL Lancaster E Stadtmauer EA Lacey SF Dengel K Ambrose DE. Posterior reversible encephalopathy syndrome (PRES) after infusion of anti-bcma CAR T cells (CART-BCMA) for multiple myeloma: Successful treatment with cyclophosphamide. Blood (2016) 128:5702. doi: 10.1182/blood.V128.22.5702.5702

[67] Pettitt D Arshad Z Smith J Stanic T Holländer G Brindley D. CAR-T cells: A systematic review and mixed methods analysis of the clinical trial landscape. Mol Ther (2018) 26:342–53. doi: 10.1016/j.ymthe.2017.10.019 PMC5835018 29248427

[68] Park JR Digiusto DL Slovak M Wright C Naranjo A Wagner J. Adoptive transfer of chimeric antigen receptor re-directed cytolytic T lymphocyte clones in patients with neuroblastoma. Mol Ther (2007) 15:825–33. doi: 10.1038/sj.mt.6300104 17299405

[69] Wang X Huynh C Urak R Weng L Walter M Lim L. The cerebroventricular environment modifies CAR T cells for potent activity against both central nervous system and systemic lymphoma. Cancer Immunol Res (2021) 9:75–88. doi: 10.1158/2326-6066.CIR-20-0236 33093217 PMC8008993

[70] Sukumar M Liu J Ji Y Subramanian M Crompton JG Yu Z. Inhibiting glycolytic metabolism enhances CD8+ T cell memory and antitumor function. J Clin Invest (2013) 123:4479–88. doi: 10.1172/JCI69589 PMC3784544 24091329

[71] Patsoukis N Bardhan K Chatterjee P Sari D Liu B Bell LN. PD-1 alters T-cell metabolic reprogramming by inhibiting glycolysis and promoting lipolysis and fatty acid oxidation. Nat Commun (2015) 6:6692. doi: 10.1038/ncomms7692 25809635 PMC4389235

[72] Theruvath J Sotillo E Mount CW Graef CM Delaidelli A Heitzeneder S. Locoregionally administered B7-H3-targeted CAR T cells for treatment of atypical teratoid/rhabdoid tumors. Nat Med (2020) 26:712–9. doi: 10.1038/s41591-020-0821-8 PMC7992505 32341579

[73] Zah E Lin MY Silva-Benedict A Jensen MC Chen YY. T Cells expressing CD19/CD20 bispecific chimeric antigen receptors prevent antigen escape by malignant b cells. Cancer Immunol Res (2016) 4:498–508. doi: 10.1158/2326-6066.CIR-15-0231 27059623 PMC4933590

[74] Schneider D Xiong Y Wu D Nölle V Schmitz S Haso W. A tandem CD19/CD20 CAR lentiviral vector drives on-target and off-target antigen modulation in leukemia cell lines. J ImmunoTher Cancer (2017) 5:42. doi: 10.1186/s40425-017-0246-1 28515942 PMC5433150

[75] Tong C Zhang Y Liu Y Ji X Zhang W Guo Y. Optimized tandem CD19/CD20 CAR-engineered T cells in refractory/relapsed b-cell lymphoma. Blood (2020) 136:1632–44. doi: 10.1182/blood.2020005278 PMC7596761 32556247

[76] Ruella M Xu J Barrett DM Fraietta JA Reich TJ Ambrose DE. Induction of resistance to chimeric antigen receptor T cell therapy by transduction of a single leukemic b cell. Nat Med (2018) 24:1499–503. doi: 10.1038/s41591-018-0201-9 PMC6511988 30275568

[77] Fernández de Larrea C Staehr M Lopez AV Ng KY Chen Y Godfrey WD. Defining an optimal dual-targeted CAR T-cell therapy approach simultaneously targeting BCMA and GPRC5D to prevent BCMA escape-driven relapse in multiple myeloma. Blood Cancer Discov (2020) 1:146–54. doi: 10.1158/2643-3230.BCD-20-0020 PMC7575057 33089218

[78] SakemuraRHefaziMSieglerELCoxMJLarsonDPHansenMJ. Targeting cancer-associated fibroblasts in the bone marrow prevents resistance to CART-cell therapy in multiple myeloma. Blood (2022) 139(26):3708–21. doi: 10.1182/blood.2021012811 35090171

[79] Spiegel JY Patel S Muffly L Hossain NM Oak J Baird JH. CAR T cells with dual targeting of CD19 and CD22 in adult patients with recurrent or refractory b cell malignancies: a phase 1 trial. Nat Med (2021) 27:1419–31. doi: 10.1038/s41591-021-01436-0 PMC8363505 34312556

[80] Shah NN Johnson BD Schneider D Zhu F Szabo A Keever-Taylor CA. Bispecific anti-CD20, anti-CD19 CAR T cells for relapsed b cell malignancies: a phase 1 dose escalation and expansion trial. Nat Med (2020) 26:1569–75. doi: 10.1038/s41591-020-1081-3 33020647

[81] Qin H Ramakrishna S Nguyen S Fountaine TJ Ponduri A Stetler-Stevenson M. Preclinical development of bivalent chimeric antigen receptors targeting both CD19 and CD22. Mol Ther Oncolytics (2018) 11:127–37. doi: 10.1016/j.omto.2018.10.006 PMC6300726 30581986

[82] Fousek K Watanabe J Joseph SK George A An X Byrd TT. CAR T-cells that target acute b-lineage leukemia irrespective of CD19 expression. Leukemia (2021) 35:75–89. doi: 10.1038/s41375-020-0792-2 32205861 PMC7519582

[83] Dai H Wu Z Jia H Tong C Guo Y Ti D. Bispecific CAR-T cells targeting both CD19 and CD22 for therapy of adults with relapsed or refractory b cell acute lymphoblastic leukemia. J Hematol Oncol (2020) 13:30. doi: 10.1186/s13045-020-00856-8 32245502 PMC7126394

[84] Xie B Li Z Zhou J Wang W. Current status and perspectives of dual-targeting chimeric antigen receptor T-cell therapy for the treatment of hematological malignancies. Cancers (2022) 14:3230. doi: 10.3390/cancers14133230 35805001 PMC9265066

[85] Wu J Meng F Cao Y Zhang Y Zhu X Wang N. Sequential CD19/22 CAR T-cell immunotherapy following autologous stem cell transplantation for central nervous system lymphoma. Blood Cancer J (2021) 11:131. doi: 10.1038/s41408-021-00523-2 34267187 PMC8282870

[86] Li T Zhao L Zhang Y Xiao Y Wang D Huang L. CAR T-cell therapy is effective but not long-lasting in b-cell lymphoma of the brain. Front Oncol (2020) 10:1306. doi: 10.3389/fonc.2020.01306 32903866 PMC7438944

[87] Tu S Zhou X Guo Z Huang R Yue C He Y. CD19 and CD70 dual-target chimeric antigen receptor T-cell therapy for the treatment of relapsed and refractory primary central nervous system diffuse Large b-cell lymphoma. Front Oncol (2019) 9:1350. doi: 10.3389/fonc.2019.01350 31867275 PMC6904344

[88] June CH O'Connor RS Kawalekar OU Ghassemi S Milone MC. CAR T cell immunotherapy for human cancer. Science (2018) 359:1361–5. doi: 10.1126/science.aar6711 29567707

[89] Xu-Monette ZY Zhou J Young KH. PD-1 expression and clinical PD-1 blockade in b-cell lymphomas. Blood (2018) 131:68–83. doi: 10.1182/blood-2017-07-740993 29118007 PMC5755041

[90] Ou A Sumrall A Phuphanich S Spetzler D Gatalica Z Xiu J. Primary CNS lymphoma commonly expresses immune response biomarkers. Neurooncol Adv (2020) 2(1):vdaa018. doi: 10.1093/noajnl/vdaa018 32201861 PMC7067145

[91] Chapuy B Roemer MG Stewart C Tan Y Abo RP Zhang L. Targetable genetic features of primary testicular and primary central nervous system lymphomas. Blood (2016) 127:869–81. doi: 10.1182/blood-2015-10-673236 PMC4760091 26702065

[92] Wang Y Wenzl K Manske MK Asmann YW Sarangi V Greipp PT. Amplification of 9p24.1 in diffuse large b-cell lymphoma identifies a unique subset of cases that resemble primary mediastinal large b-cell lymphoma. Blood Cancer J (2019) 9:73. doi: 10.1038/s41408-019-0233-5 31471540 PMC6717207

[93] Takashima Y Kawaguchi A Sato R Yoshida K Hayano A Homma J. Differential expression of individual transcript variants of PD-1 and PD-L2 genes on Th-1/Th-2 status is guaranteed for prognosis prediction in PCNSL. Sci Rep (2019) 9:10004. doi: 10.1038/s41598-019-46473-5 31292525 PMC6620277

[94] Qiu Y Li Z Pouzoulet F Vishnu P Copland JA 3rd Knutson KL. Immune checkpoint inhibition by anti-PDCD1 (anti-PD1) monoclonal antibody has significant therapeutic activity against central nervous system lymphoma in an immunocompetent preclinical model. Br J Haematol (2018) 183:674–8. doi: 10.1111/bjh.15009 29076134

[95] Nayak L Iwamoto FM LaCasce A Mukundan S Roemer MGM Chapuy B. PD-1 blockade with nivolumab in relapsed/refractory primary central nervous system and testicular lymphoma. Blood (2017) 129:3071–3. doi: 10.1182/blood-2017-01-764209 PMC5766844 28356247

[96] Furuse M Nonoguchi N Omura N Shirahata M Iwasaki K Inui T. Immunotherapy of nivolumab with dendritic cell vaccination is effective against intractable recurrent primary central nervous system lymphoma: A case report. Neurol Med Chir (Tokyo) (2017) 57:191–7. doi: 10.2176/nmc.cr.2016-0330 PMC5409273 28331101

[97] Dandachi D Ostrom QT Chong I Serpa JA Giordano TP Kruchko C. Primary central nervous system lymphoma in patients with and without HIV infection: a multicenter study and comparison with U. S Natl data. Cancer Causes Control (2019) 30:477–88. doi: 10.1007/s10552-019-01144-8 30888569

[98] Abramson JS Irwin KE Frigault MJ Dietrich J McGree B Jordan JT. Successful anti-CD19 CAR T-cell therapy in HIV-infected patients with refractory high-grade b-cell lymphoma. Cancer (2019) 125:3692–8. doi: 10.1002/cncr.32411 31503324

[99] Roberts MR Qin L Zhang D Smith DH Tran AC Dull TJ. Targeting of human immunodeficiency virus-infected cells by CD8+ T lymphocytes armed with universal T-cell receptors. Blood (1994) 84:2878–89. doi: 10.1182/blood.V84.9.2878.2878 7949163

[100] Deeks SG Wagner B Anton PA Mitsuyasu RT Scadden DT Huang C. A phase II randomized study of HIV-specific T-cell gene therapy in subjects with undetectable plasma viremia on combination antiretroviral therapy. Mol Ther (2002) 5:788–97. doi: 10.1006/mthe.2002.0611 12027564

[101] Liu B Zhang W Xia B Jing S Du Y Zou F. Broadly neutralizing antibody-derived CAR T cells reduce viral reservoir in individuals infected with HIV-1. J Clin Invest 131 (2021) 131(19):e150211. doi: 10.1172/JCI150211 PMC8483761 34375315

[102] Jiang Z Liang H Pan H Liang Y Wang H Yang X. HIV-1-Specific CAR-T cells with cell-intrinsic PD-1 checkpoint blockade enhance anti-HIV efficacy. Front Microbiol (2021) 12:684016. doi: 10.3389/fmicb.2021.684016 34295319 PMC8290485

[103] Leibman RS Richardson MW Ellebrecht CT Maldini CR Glover JA Secreto AJ. Supraphysiologic control over HIV-1 replication mediated by CD8 T cells expressing a re-engineered CD4-based chimeric antigen receptor. PloS Pathog (2017) 13:e1006613. doi: 10.1371/journal.ppat.1006613 29023549 PMC5638568

[104] Liu B Zou F Lu L Chen C He D Zhang X. Chimeric antigen receptor T cells guided by the single-chain fv of a broadly neutralizing antibody specifically and effectively eradicate virus reactivated from latency in CD4+ T lymphocytes isolated from HIV-1-Infected individuals receiving suppressive combined antiretroviral therapy. J Virol (2016) 90:9712–24. doi: 10.1128/JVI.00852-16 PMC5068523 27535056

[105] Hale M Mesojednik T Romano Ibarra GS Sahni J Bernard A Sommer K. Anti-HIV chimeric antigen receptor T cells. Mol Ther (2017) 25:570–9. doi: 10.1016/j.ymthe.2016.12.023 PMC5363191 28143740

[106] Cook MR Dorris CS Makambi KH Luo Y Munshi P Donato M. Toxicity and efficacy of CAR T-cell therapy in PCNSL and SCNSL: A meta-analysis of 128 patients. Blood Adv (2022). doi: 10.1182/bloodadvances.2022008525 PMC9813524 36260735

